# Digestive Properties of the Gastric Fluid

**Published:** 1845-05

**Authors:** 


*Digestive properties of the gastric fluid.*—Some singular evidence of these is furnished by MM. Bernard and Barreswill who have found that nutritive substances injected in simple aqueous solution into the blood are not assimulated: but are assimulated if dissolved by the aid of the gastric fluid. Among other experiments are these: portions of cane sugar, albumen, and gelatine, seven and a half grains of each, and severally dissolved in water, were injected into the jugular veins of three dogs. Three hours after, the urine of each was examined, and in each the injected substance was found. Under other similar conditions, the same quantities of the same substances dissolved in gastric fluid were injected, and three hours after, gelatine was detected in the urine of the dog into whom it had been injected, but not a trace of albumen or sugar in the urine of either of the others. Three dogs were then fed exclusively and respectively on gelatine, albumen, and sugar; and the first alone could ever be detected in the urine. The authors fed themselves in the same way and obtained the same result; and they conclude (as others presently to be mentioned do) that gelatine is not assimilable and therefore not nutritive.

Among the experiments which M. Blondlot made to determine the mode in which the gastric fluid, in or out of the stomach, acts on different animal substances some afforded novel and interesting results; A. He shows that coagulated albumen owes its long resistance to the digestive fluid only to its compact form. When coagulated in very fine particles (as by pouring white of egg beaten into a froth, into boiling water) it is digested as quickly as soft fibrine. B. He adds further evidence that the action of the stomach in coagulating milk is not due peculiarly to its digestive principle, but to its acid, which acts like the lactic acid developed from the sugar under the influence of rennet or any other decomposing azotized compound: C. The effect of the gastric fluid on bones, observed both on the bones in their entire state, and on their animal and inorganic constituents separately, is, that first, it very slowly disintegrates the animal matter, attacking, them from the surface, and then, also very slowly, disintegrates and reduces the earthy matter into a fine chalky powder, but without either dissolving or decomposing it. The earthy matter not being dissolved proves that no hydrchloric acid had acted upon it: and in its minutely divided state it all passes through the intestines and is discharged with the fæces.

The result of many more of his experiments of this kind are interesting. They confirm Mr. Beaumont's, and appeared to M. Blondlot to show that of all the simple alimentary substances, those which are fluid at the ordinary temperaature of the stomach, and those which are easily soluble (in the ordinary manner of solution) in its secretion, such as fluid albumen, sugar, gum, pectine, &c., are at once absorbed by the veins; and that others, which are not liquid nor easily soluble, such as fibrine, coagulated vegetable and animal albumen, caseine, gelatine, &c., are, in only a very small proportion, if at all, dissolved, the action of the gastric fluid on them being limited to the softening of them, so that they are reduced into very minute particles which (out of the stomach) appear like a very fine precipitate. The same general rule is said to be observed in the digestion of the compound alimentary substances, both animal and vegetable; the fluid and easily soluble parts cannot be said to be digested, for they are at once absorbed by the stomach; the rest are softened and reduced into very minute particles, which are carried into the intestines, without any change in their chemical constitution, and are, in this state absorbed by open mouths of the lacteals, visible with the naked eye, at the extremities of the villi(!) This act of softening is, in some cases, due merely to the acid of the gastric fluid: e. g. in the case of parenchymatous tissues, and succulent fruits and roots, which are similarly softened, at the same temperature, in acidulated water; in the cases of fibrine, coagulated albumen, &c., it is the effect of the *peculiar* mode of action of the gastric fluid.

In any case, chymification is, in M. Blondlot's opinion, no *solution*, but a *division* of the aliment; it undergoes no kind of decomposition.

